# Diffusion-weighted imaging and diffusion kurtosis imaging for early evaluation of the response to docetaxel in rat epithelial ovarian cancer

**DOI:** 10.1186/s12967-018-1714-1

**Published:** 2018-12-05

**Authors:** Su-juan Yuan, Tian-kui Qiao, Jin-Wei Qiang

**Affiliations:** 1 Department of Oncology, Jinshan Hospital, Shanghai Medical College, Fudan University, 1508 Longhang Road, Shanghai, 201508 People’s Republic of China; 2 Department of Radiology, Jinshan Hospital, Shanghai Medical College, University, 1508 Longhang Road, Shanghai, 201508 People’s Republic of China

**Keywords:** Epithelial ovarian cancer, Diffusion-weighted imaging, Diffusion kurtosis imaging, Ki-67, Cancer antigen 125, Docetaxel, Apoptosis, Tumor necrosis

## Abstract

**Background:**

To investigate diffusion-weighted magnetic imaging (DWI) and diffusion kurtosis magnetic imaging (DKI) for the early detection of the response to docetaxel (DTX) chemotherapy in rat epithelial ovarian cancer (EOC).

**Methods:**

7,12-Dimethylbenz[A]anthracene was applied to induce orthotopic EOC in Sprague–Dawley rats. Rats with EOC were treated with DTX on day 0 (treatment group) or were left untreated (control group). DWI and DKI were performed on days 0, 3, 7, 14 and 21 after treatment. On day 21, the tumors were categorized into the sensitive and insensitive groups according to the size change. The cutoff values of the DWI and DKI parameters for the early response were determined. The experiment was repeated, and the treatment group was divided into the sensitive and insensitive groups according to the initially obtained cutoff values. The DWI and DKI parameters were correlated with tumor size, proliferation, apoptosis and tumor necrosis.

**Results:**

In the sensitive* vs.* insensitive or control group, significant differences were found in the Δ% of the DWI and DKI parameters (ADC, D and K) from day 3 and in tumor size from day 14. Early on day 7, the Δ% of K had an AUC of 1 and sensitivity and specificity values of 100% and 100%, respectively, to detect the response to DTX using a cutoff value of 19.03% reduction in K. From day 7, significant differences were found in the Δ% of Ki-67 and CA125 in the sensitive *vs*. control group and from day 14 in the sensitive vs. insensitive group. From day 14, there were significant differences in the Δ% of Bcl-2, apoptosis and tumor necrosis in the sensitive *vs*. control or insensitive group. The Δ% values of ADC and D were negatively correlated with the Δ% values of tumor size, Ki-67, CA125 and Bcl-2 and were positively correlated with the Δ% values of apoptosis and tumor necrosis. The Δ% of K was positively correlated with the Δ% values of tumor size, Ki-67, CA125 and Bcl-2 and was negatively correlated with the Δ% values of apoptosis and tumor necrosis.

**Conclusions:**

DWI and DKI parameters, especially K, are superior for imaging tumor size for the early detection of the response to DTX chemotherapy in induced rat EOC.

## Background

Ovarian cancer is one of the most common malignant tumors in female genital organs, and the mortality rate ranks first in gynecological tumors. Epithelial ovarian cancer (EOC) is the most common histological type, accounting for approximately 90% of malignant ovarian tumors. Despite the development of cytoreductive surgery and chemotherapy,the overall 5-year survival rate of EOC patients remains approximately 40% [1]. Therefore, developing new therapeutic approaches is imperative. Since the 1990s, docetaxel plus platinum has become the first-line chemotherapeutic regimen of EOC [2]. Although proven effective for EOC, DTX benefits only some patients because of resistance or insensitivity. For non-responders, the traditional response evaluation criteria in solid tumors (RECIST) based on changes in tumor size as well as the marker levels cannot reflect the microstructural changes in the tumor after chemotherapy sufficiently early. Therefore, developing a reliable method to detect a response as early as possible will be significant to avoid noneffective chemotherapy and wasted time.

Magnetic resonance (MR) diffusion-weighted imaging (DWI) offers monitoring prior to morphological changes. Chemotherapy drugs contribute to kill tumor cells, subsequently decreasing the cell density and restricted diffusion motions of water molecules. The study of colorectal cancer by Delli et al. demonstrated that the increased apparent diffusion coefficient (ADC) value of a tumor after chemotherapy was negatively correlated with tumor cell proliferation and was positively correlated with apoptosis [3]. In addition, many studies have demonstrated that DWI is an important biomarker in the prediction and early evaluation of the efficacy of treatment for EOC [4–7]. Diffusion kurtosis imaging (DKI) is an advanced technique based on a non-Gaussian model and can reflect water diffusion deviation from Gaussian behavior [Jensen et al.]. Compared with standard DWI, it can more accurately describe the complicated water diffusivity in biological tissue and provide more information about tissue heterogeneity and cellularity with parameters of D (corrected ADC) and K (diffusion kurtosis) [8]. DKI has mainly been used for the differential diagnosis of benign and malignant tumors [9, 10]. Until now, to the best of our knowledge, DKI has not been applied for the early evaluation and prediction of the chemotherapy response of EOC, except in a few studies concerning glioblastoma and nasopharyngeal carcinoma [11–13]. Therefore, the objective of our study was to investigate whether the ADC value of DWI, and K and D values of DKI can be used to assess the early response to DTX chemotherapy in induced rat EOC by correlating with the tumor size, Ki-67, Bcl-2, apoptosis and tumor necrosis.

## Methods

### Tumor model

All animal experimental procedures were approved by Institutional Animal Care committee of Jinshan Hospital of Fudan University and were performed according to the Guide for the Care and Use of Laboratory Animals of the National Science and Technology Committee of China. Two hundred female Sprague–Dawley (SD) rats (8 weeks old; Shanghai Laboratory Animal Research Center, Shanghai, China) underwent surgery to establish orthotopic EOCs. The surgical procedures and protocol of the induction of EOCs were the same as those in our previous study [14]. After rat was anesthetized, the right ovary was surgically exposed, packed with a piece of absorbable cloth (0.5 cm × 0.5 cm) coating 2 mg carcinogen 7,12-Dimethylbenz[A]anthracene (DMBA). Twenty rats died in the process of surgery and tumorigenesis. In the remaining 180 rats, 170 rats developed ovarian malignancies. Among them, 138 rats with tumors were randomly selected into our experiment and 117 EOCs were designed into the analysis of our study after excluding 21 non-EOCs.

### Experimental design

**Fig. 1:**
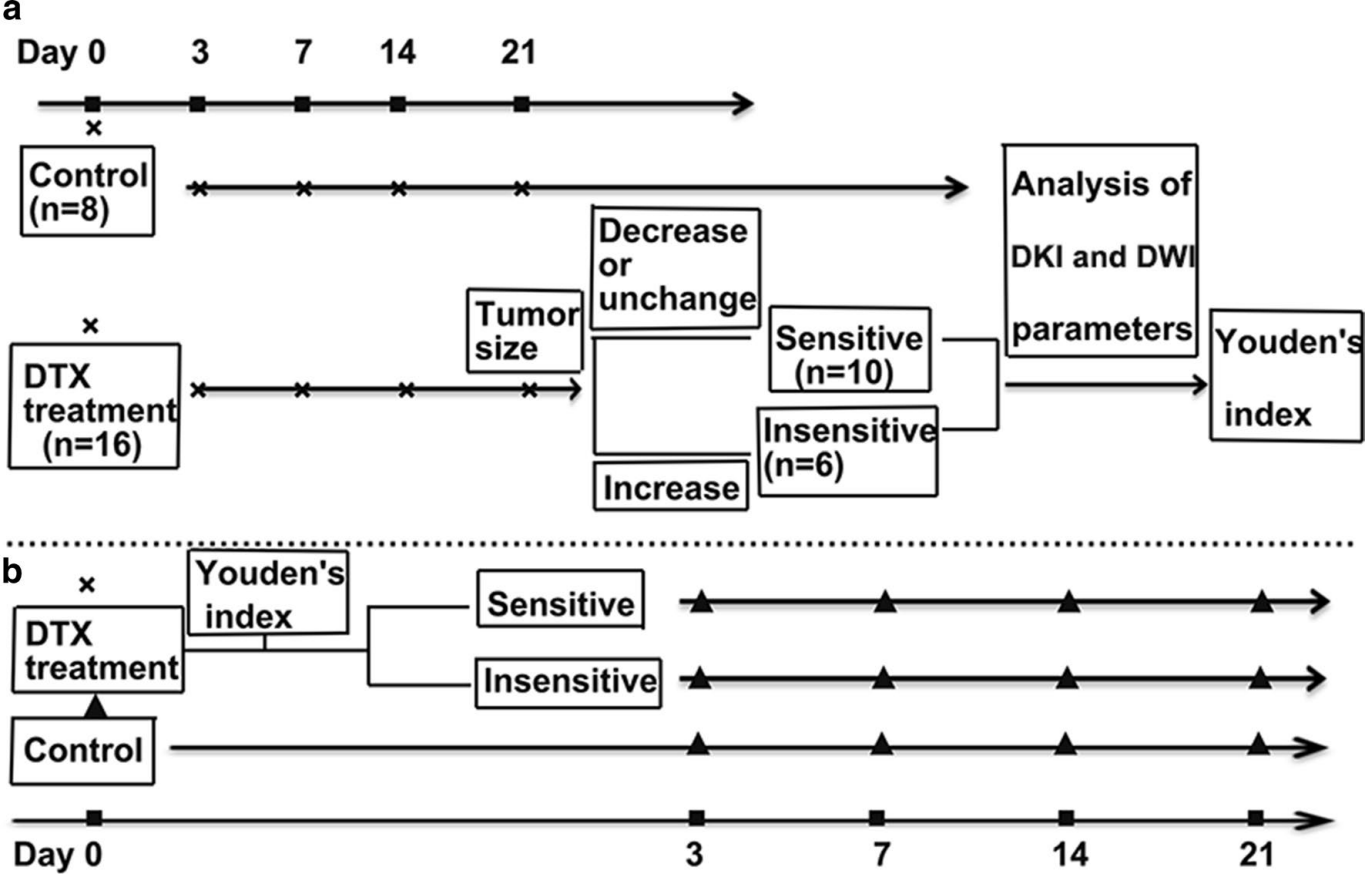
Flow chart of the study design showing grouping, treatment and imaging time points. **a** DWI and DKI  were performed for all rats on day 0 after which they were randomly assigned to the treatment and control groups. The rats in the treatment group received 12 mg/kg of DTX. Before (day 0) and 3, 7,14, 21 days after DTX therapy, all rats underwent MRI scanning. Twenty-one days after DTX therapy, the tumors were categorized into the sensitive group and the insensitive group. Youden’s index was obtained using retrospective logistic regression analysis of the DWI and DKI parameters at different time points. **b** Another 93 rats received the same DTX, DWI and DKI as those in **a**. According to the above cutoff value at the optimal time point, the treatment rats were categorized into the sensitive or insensitive groups. Several rats in the control group on day 0 and in every group at different time points were killed, and the tumors were excised for histology (“black up-pointing triangle” indicated DWI, DKI and histology). Δ% DWI/DKI parameter = (DWI/DKI parameter_after_ − DWI/DKI parameter_day 0_)/DWI/DKI parameter_day 0_ × 100%. Δ% tumor size = (tumor size_after_ − tumor size_day 0_)/tumor size_day 0_ × 100%. Δ% Ki-67/Bcl-2/apoptosis rate/necrosis rate = (Ki-67/Bcl-2/apoptosis rate/necrosis rate _after_ − Ki-67/Bcl-2/apoptosis rate/necrosis rate_day 0_)/Ki-67/Bcl-2/apoptosis rate/necrosis rate_day 0_ × 100%. (Note: The tumor size indicated the longest diameter of the tumor; Ki-67_day 0_, Bcl-2_day 0_, apoptosis rate_day 0_ and necrosis_day 0_ represented the average expression of corresponding biomarkers in the control group on day 0)

The study consisted of training and validation parts (Fig. 1). In the training part (a), 180 days after operation, 24 rats were randomly assigned to treatment (n = 16) and control (n = 8) groups. In the treatment rats, 12 mg/kg of DTX was administered via the caudal vein [15]. All rats underwent standard DWI and DKI scanning before (day 0) and on the 3rd, 7th, 14th, and 21st days after DTX therapy. On day 21 after DTX therapy, rats with EOCs were divided into the sensitive group (tumor with decreased or unchanged size) and insensitive group (tumor with increased size) by referencing the RECIST guidelines and a previous study [16, 17]. DWI and DKI parameters and tumor sizes at different time points were analyzed retrospectively, and Youden’s index, the cutoff value that represents predictive factors to assess an early therapy response, was obtained using logistic regression analysis and receiver operating characteristic (ROC) curve analysis. In the validation part (b), the experiment was repeated, and rats received the same DTX therapy and MRI scanning as those in part A. The treatment group was divided into the sensitive and insensitive groups at an optimal time point according to the obtained Youden’s index. Several rats in every group at different time points and in the control group on day 0, respectively, were killed for histopathological and immunohistochemical (IHC) analyses. In both parts, at different time points, only rats with EOCs demonstrated by histopathology were included in the study. In part B, the number of rats with EOCs in different groups and at different time points are listed in Table 1.

**Table 1:** Number of rats with EOC in different groups and at different time points

Groups	D0	D3	D7	D14	D21
Treatment group	48				
Sensitive group	30 (0)	30 (0)	30 (10)	20 (9)	11 (11)
Insensitive group	18 (0)	18 (0)	18 (7)	11 (56)	5 (5)
Control group	45 (9)	36 (9)	27 (10)	17 (8)	9 (9)

The number of rats sacrificed for histopathology is in parentheses

### MRI scanning

Rats underwent MRI scanning under anesthesia using a 3.0 T scanner (Verio; Siemens Healthcare, Erlangen, Germany) with a rat coil. The following sequences were obtained: axial spin echo (SE) T1-weighted imaging (T1WI) (time of repetition/time of echo [TR/TE] = 7.29/2.28 ms); axial, sagittal and coronal turbo SE T2W1 with fat saturation [TR/TE = 2500/93 ms]; and turbo SE T2W1 (TR/TE = 8000/98 ms). DWI and DKI were performed using a single-shot echo planar imaging (EPI) sequence. The imaging parameters were: TR/TE, 3300/71 ms; section thickness, 2 mm; field of view (FOV), 120 × 64.9 mm^2^; matrix, 148 × 148; 4 b factors of DKI: 0, 700, 1400, 1500, 2100 s/mm^2^; and acquisition time, 315 s. 2 b factors of DWI 0 and 1000 s/mm^2^.

### DWI and DKI processing

The DKI and DWI images were independently evaluated by two radiologists with 10 years of experience in pelvic MRI and who were blinded to the histopathological information. The non-Gaussian DKI model was mathematically expressed by the following nonlinear equation: S_b_ = S_0_·exp(−b·D + 1/6b^2^·D^2^·K), in which S_b_ is the DWI signal intensity at a particular b value, and S_0_ is the signal intensity when the b value is 0 s/mm^2^. D represents the corrected ADC of the non-Gaussian distribution behavior, and K is the apparent diffusional kurtosis, a dimensionless parameter that reflects the deviation of diffusion from Gaussian behavior. D and K were calculated using in-house software (MatLab; MathWorks, Natick, MA, USA). ADC maps were generated from the standard DWI with b values of 0 and 1000 s/mm^2^.

### Histopathological and IHC analysis

In the validation part (b), after completing MRI scanning at every time point, the rat ovary was removed and fixed for HE staining to evaluate histopathology and tumor necrosis, and for IHC staining to investigate the expression of Ki-67 and Bcl-2, as previously described [14]. Tumor necrosis was semi-quantitatively analyzed using Image-Pro Plus 6.0 imaging software [18]. Tumor necrosis rate = necrosis area/field area × 100%. The tumor necrosis, Ki-67 and Bcl-2 statuses in three randomly selected high-power fields (HPF × 200) were obtained and averaged.

### ELISA

At the target time point, the evaluation of cancer antigen 125 (CA125) in murine serum was applied with ELISA as previously described [14].

### Flow cytometry analysis

EOC tissues were made into single cell suspension that were incubated with FITC-conjugated Annexin-V and propidium iodide for 15 min at room temperature. Immediately, the cells were analyzed using a Cytomics™ FC500 flow cytometer (Beckman Coulter, Inc., Fullerton, CA, USA). The data were analyzed using Summit version 5.2 software (Beckman Coulter, Inc.). Apoptosis rate = number of pieces of apoptosis cells/total number of tumor cells.

### Statistical analysis

The data were analyzed using SPSS 22.0 (Chicago, IL, USA) and are presented as the mean ± standard deviation. One-way analysis of variance (ANOVA) was used for data analysis between multiple groups, and the differences between every two group were analyzed by the least significant difference test (LSD-t). Spearman’s correlation was used to analyze the correlation between the change rates of DWI and DKI parameters and change rates of the tumor size, necrosis, apoptosis, Ki-67 and Bcl-2. The results were interpreted according to the degree of association as strong (1 ≥ r ≥ 0.75), moderate (0.75 > r ≥ 0.50), low (0.50 > r ≥ 0.25), or not relevant (r < 0.25) after taking significant correlation (*p* < 0.01 or *p* < 0.05) values into consideration [19–21]. A *p* < 0.05 was considered statistically significant.

## Results

### Therapeutic effect of DTX for rat EOC

**Fig. 2:**
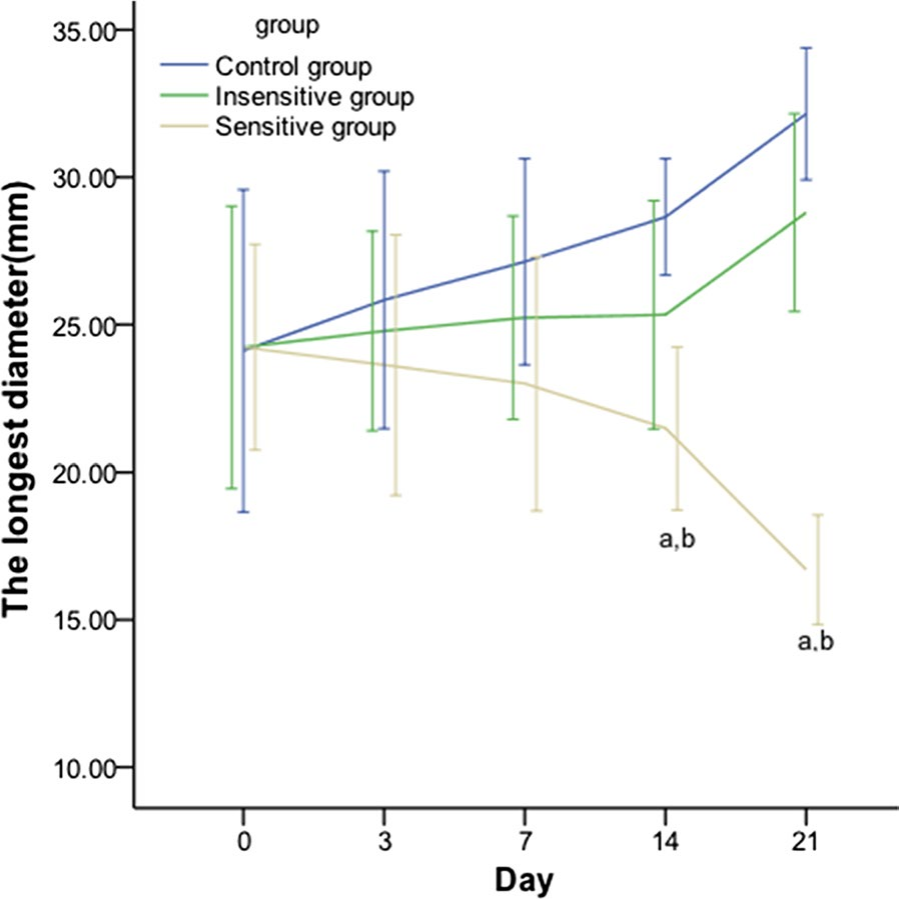
Line graph showing the effect of DTX on the tumor size in rat EOCs. From day 14 after DTX administration, the tumor size of the sensitive group was significantly decreased compared with that of the insensitive and control counterparts. The tumor size indicates the longest diameter. *p *< 0.05, ^a^:sensitive vs. control; ^b^: sensitive vs. insensitive

According to the results of training part (A), on days 0, 3 and 7, the difference in the mean tumor size of EOCs among the three groups was not significant; from day 14 after DTX administration, the tumor size of the sensitive group was significantly decreased compared with those of the insensitive and control counterparts (Fig. 2). Differences were found in the Δ% of the tumor size in the sensitive *vs.* control group from day 7 and in the sensitive *vs.* insensitive group from day 14. No differences were found between the insensitive and control groups at all time points (Table 2).

**Table 2:** Change rates in the tumor size at different time points (%)

Group	Day 3	Day 7	Day 14	Day 21
Sensitive	− 2.17 ± 5.35	− 6.27 ± 3.80^a^	− 16.69 ± 2.96^a,b^	− 30.46 ± 3.05^a,b^
Insensitive	4.09 ± 6.69	4.85 ± 3.72	10.57 ± 5.61	20.58 ± 4.24
Control	8.53 ± 4.33	14.37 ± 4.543	21.11 ± 4.67	37.49 ± 9.64

Tumor size: longest diameter

*p *< 0.05

^a^Sensitive vs. control; ^b^ Sensitive vs. insensitive

### Changes in the DWI and DKI parameters after DTX therapy in rat EOC

**Fig. 3:**
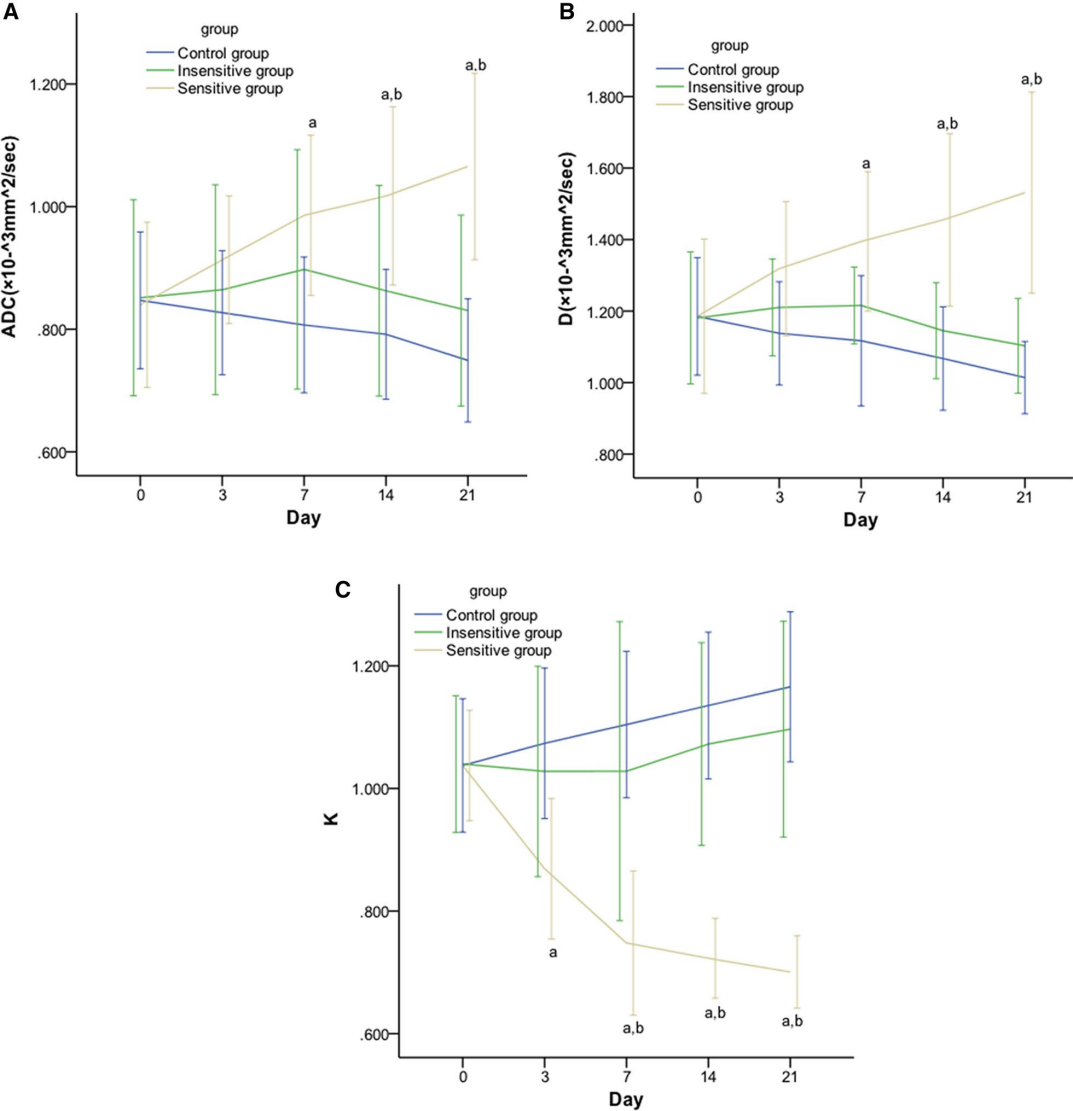
Line graphs showing the effect of DTX on DWI and DKI parameters in rat EOCs. From day 7 after DTX administration, ADC (**A**) and D (**B**) values of the sensitive group were significantly decreased compared with those of the insensitive and control counterparts from day 14. K (**C**) value of the sensitive group is significantly decreased compared to its insensitive counterpart from day 3 and to control group from day 14. *p *< 0.05, ^a^: sensitive vs. control; ^b^: sensitive vs. insensitive

**Fig. 4:**
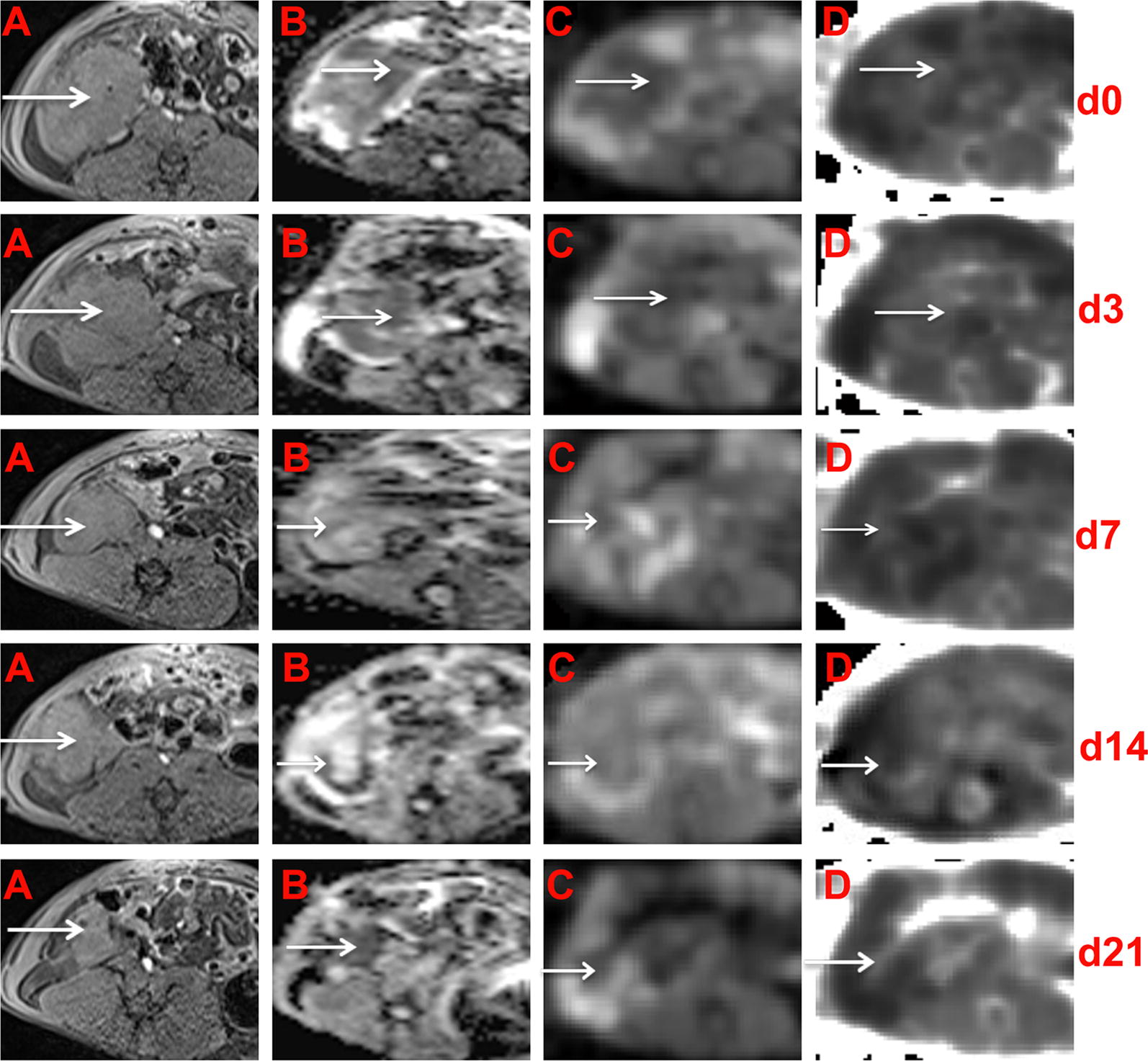
Conventional, DWI and DKI of EOCs in sensitive rats at different time points. In the right adnexal area, a multilocular cystic-solid EOC (arrow) progressively decreased on T1WI (**A**). ADC maps were generated from standard DWI (**B**) and D maps (**C**) and K (**D**) maps were generated from DKI. The solid component) showed hypointensity on the ADC maps, hyperintensity on the K maps and hypointensity on the D maps

**Fig. 5:**
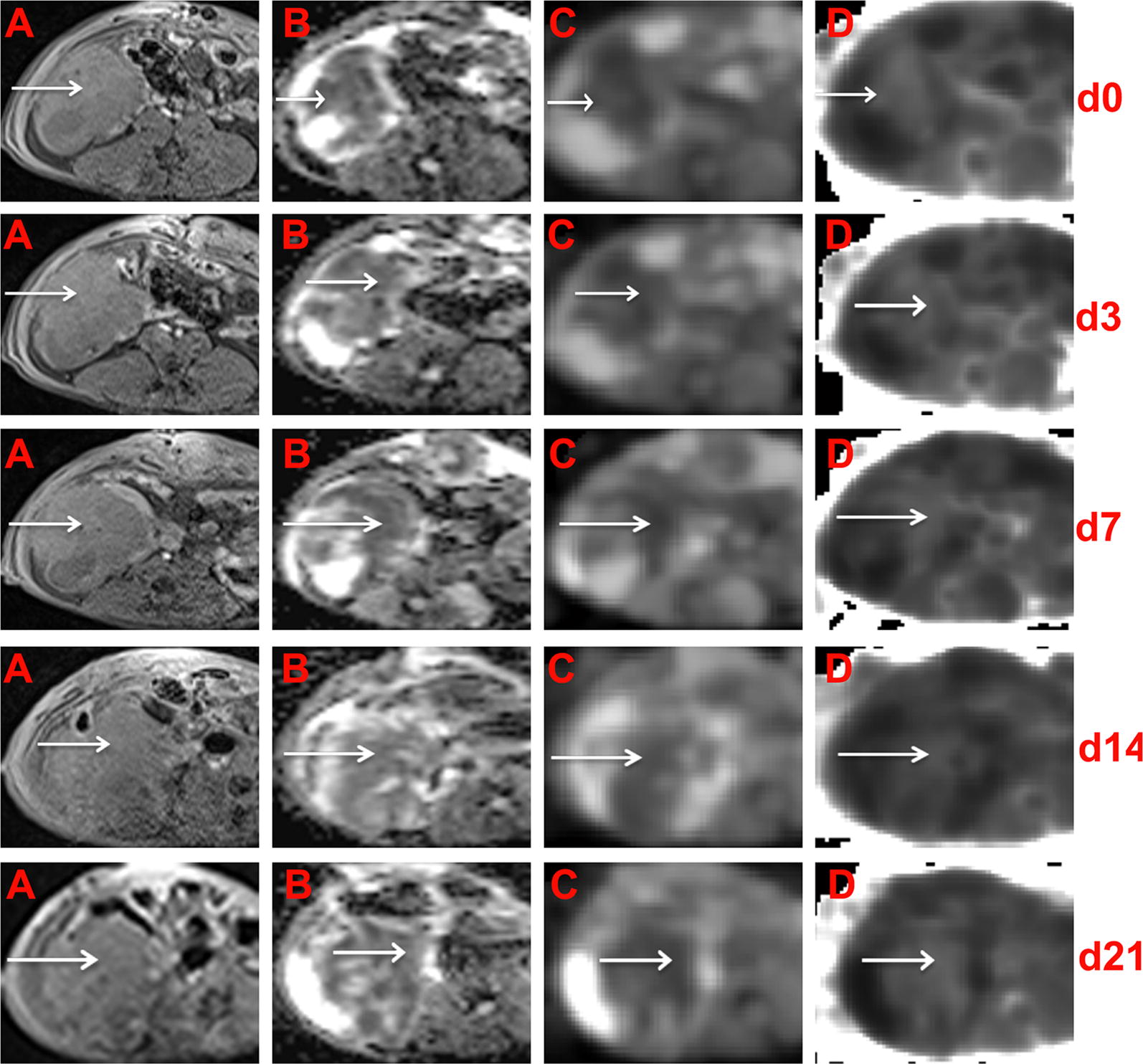
Conventional, DWI and DKI of EOCs in sensitive rats at different time points. In the right adnexal area, a multilocular cystic-solid EOC (arrow) progressively increased on T1WI (**A**). ADC maps were generated from standard DWI (**B**), and D maps (**C**) and K (**D**) maps were generated from DKI. The solid component showed hypointensity on the ADC maps, hyperintensity on the K maps and hypointensity on the D maps

On days 0, ADC of DWI, and D and K of DKI in rat EOC showed no significant differences among the three groups. On day 3, there was a significant difference in K in the sensitive vs. control group. On day 7, significant differences were found in K in the sensitive vs. control or insensitive group, and in ADC and D in the sensitive vs. control group. On days 14 and 21, significant differences were found in the pairwise comparisons, except for ADC and D in the insensitive vs. control group (Fig. 3). On days 3, 7, 14 and 21, significant differences were found in the pairwise comparisons in the Δ% values of all three parameters (ADC, D and K), except for ADC, D and K in the insensitive vs. control group (Table 3, Figs. 4, 5).

**Table 3:** Change rates of DWI and DKI parameters at different time points (%)

Time	Group	Δ% ADC	Δ% D	Δ% K
Day 3	Sensitive	20.55 ± 2.40^a,b^	22.44 ± 5.65^a,b^	− 16.28 ± 2.99^a,b^
	Insensitive	1.39 ± 1.54	3.16 ± 2.89	− 1.61 ± 2.24
	Control	− 2.22 ± 1.85	− 3.81 ± 1.63	3.42 ± 0.95
Day 7	Sensitive	17.94 ± 2.61^a,b^	18.55 ± 3.48^a,b^	− 28.22 ± 1.95^a,b^
	Insensitive	5.09 ± 3.28	4.01 ± 3.87	− 2.14 ± 4.90
	Control	− 4.57 ± 3.31	− 5.81 ± 2.94	6.42 ± 1.07
Day 14	Sensitive	21.67 ± 3.14^a,b^	23.39 ± 5.36^a,b^	− 30.32 ± 0.57^a,b^
	Insensitive	1.50 ± 4.22	− 2.34 ± 3.62	2.80 ± 2.50
	Control	− 6.37 ± 2.94	− 9.78 ± 2.47	9.48 ± 1.84
Day 21	Sensitive	27.41 ± 3.56^a,b^	29.33 ± 3.70^a,b^	− 32.43 ± 1.13^a,b^
	Insensitive	− 2.18 ± 3.78	− 6.05 ± 2.87	5.09 ± 2.97
	Control	− 11.38 ± 2.63	− 13.75 ± 3.60	12.42 ± 2.02

Δ%: change rate

*p *< 0.05

^a^Sensitive vs. control

^b^Sensitive vs. insensitive

### ROC evaluation based on DWI and DKI parameters

On days 0 and 3, the ADC, D and K could not evaluate the response to DTX therapy in rat EOC. K showed its ability to evaluate the efficacy of DTX in EOC, with AUC, sensitivity and specificity values of 0.917, 83.3% and 83.3%, respectively, on day 7, and values of 1, 100% and 100%, respectively, on day 14. However, early on day 7, the change rate of K showed perfect ability to evaluate the efficacy of DTX in EOCs, with AUC, sensitivity and specificity values of 1, 100% and 100%, respectively. Youden’s index was a 19.03% reduction in K.

### Effective DTX therapy decreases Ki-67 expression and the CA125 level in rat EOC

**Fig. 6:**
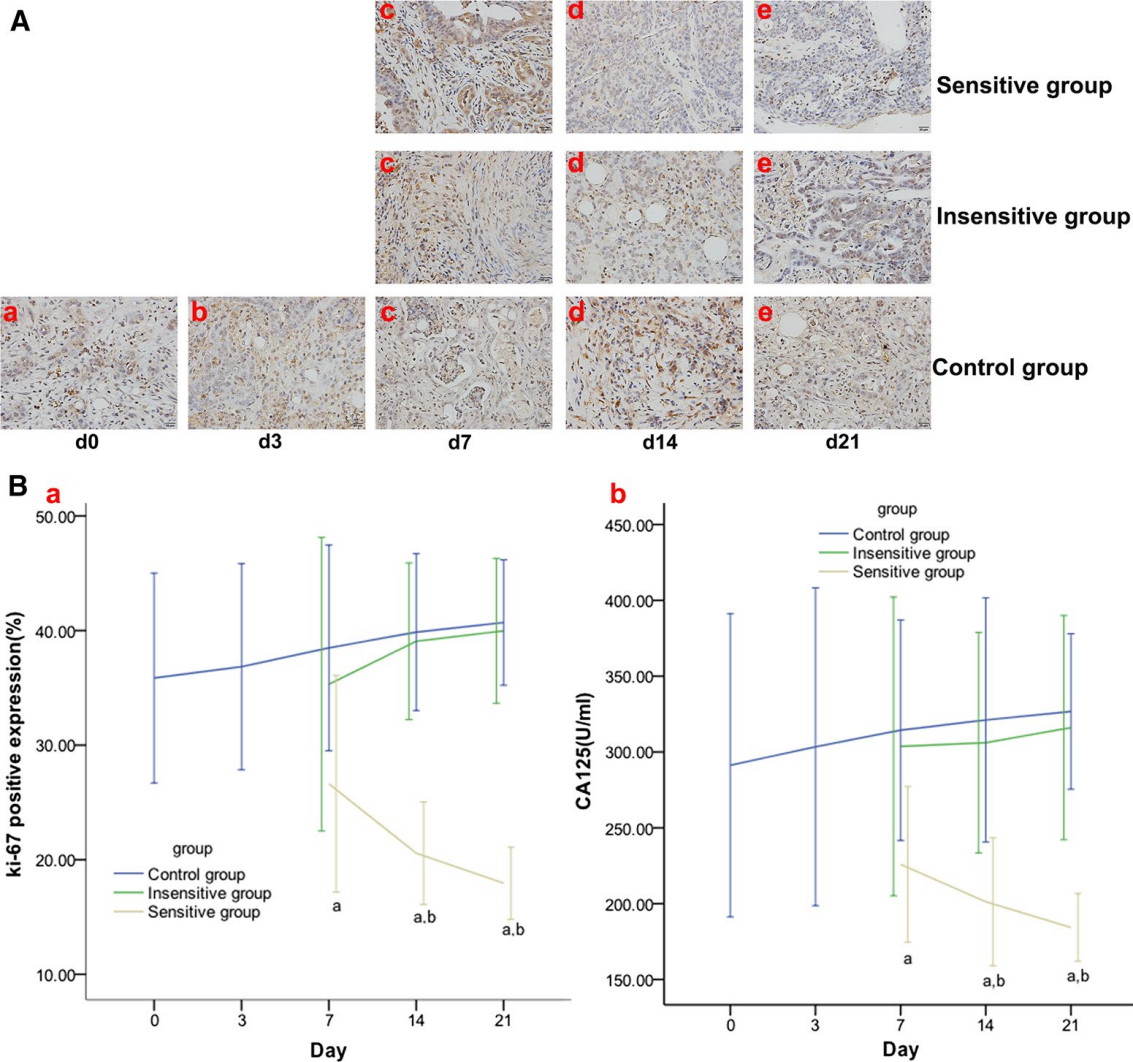
Effect of DTX on Ki-67 and CA125 in rat EOCs. IHC staining (HRF × 200) on day 0 (a), day 3 (b), day 7 (c), day 14 (d), and day 21 (e) of Ki-67 (**A**), which manifest as yellow or brown granules and appear in Ki-67-positive cell nuclei were analyzed. The Ki-67 expression and CA125 level (**B**) were decreased in the sensitive group but increased in the insensitive or control group. From day 7, significant differences were found in Ki-67 and CA125 in the sensitive vs. control group and in the sensitive vs. insensitive group from day 14. *p *< 0.05, ^a^: sensitive vs. control; ^b^: sensitive vs. insensitive

According to the results of validation part (B), on day 7, the treatment group was divided into the sensitive and insensitive groups, with a 19.03% reduction in K. Ki-67 expression and the serum CA125 level, and the Δ% of Ki-67 expression and serum CA125 level were significantly different between the sensitive and control groups on day 7 and were significantly different between the sensitive vs control or insensitive group on days 14 and 21 (Fig. 6 and Table 4).

**Table 4:** Change rates of Ki-67 expression and the serum CA125 level at different time points (%)

Time	Group	Δ% Ki-67	Δ% CA125
Day 7	Sensitive group	− 25.75 ± 10.26^a^	− 22.45 ± 10.26^a^
	Insensitive group	− 1.52 ± 11.22	4.27 ± 10.63
	Control group	7.32 ± 9.01	7.94 ± 8.99
Day 14	Sensitive group	− 42.65 ± 4.86^a,b^	− 30.90 ± 5.64^a,b^
	Insensitive group	8.94 ± 6.00	5.11 ± 7.84
	Control group	11.16 ± 6.87	10.24 ± 9.96
Day 21	Sensitive group	− 49.93 ± 3.41^a,b^	− 36.71 ± 2.99^a,b^
	Insensitive group	11.47 ± 5.54	8.51 ± 7.97
	Control group	13.50 ± 5.49	12.18 ± 6.34

Δ%: change rate

*p *< 0.05

^a^Sensitive vs. control

^b^Sensitive vs. insensitive

### Effective DTX therapy decreases Bcl-2 expression in rat EOCs

**Fig. 7:**
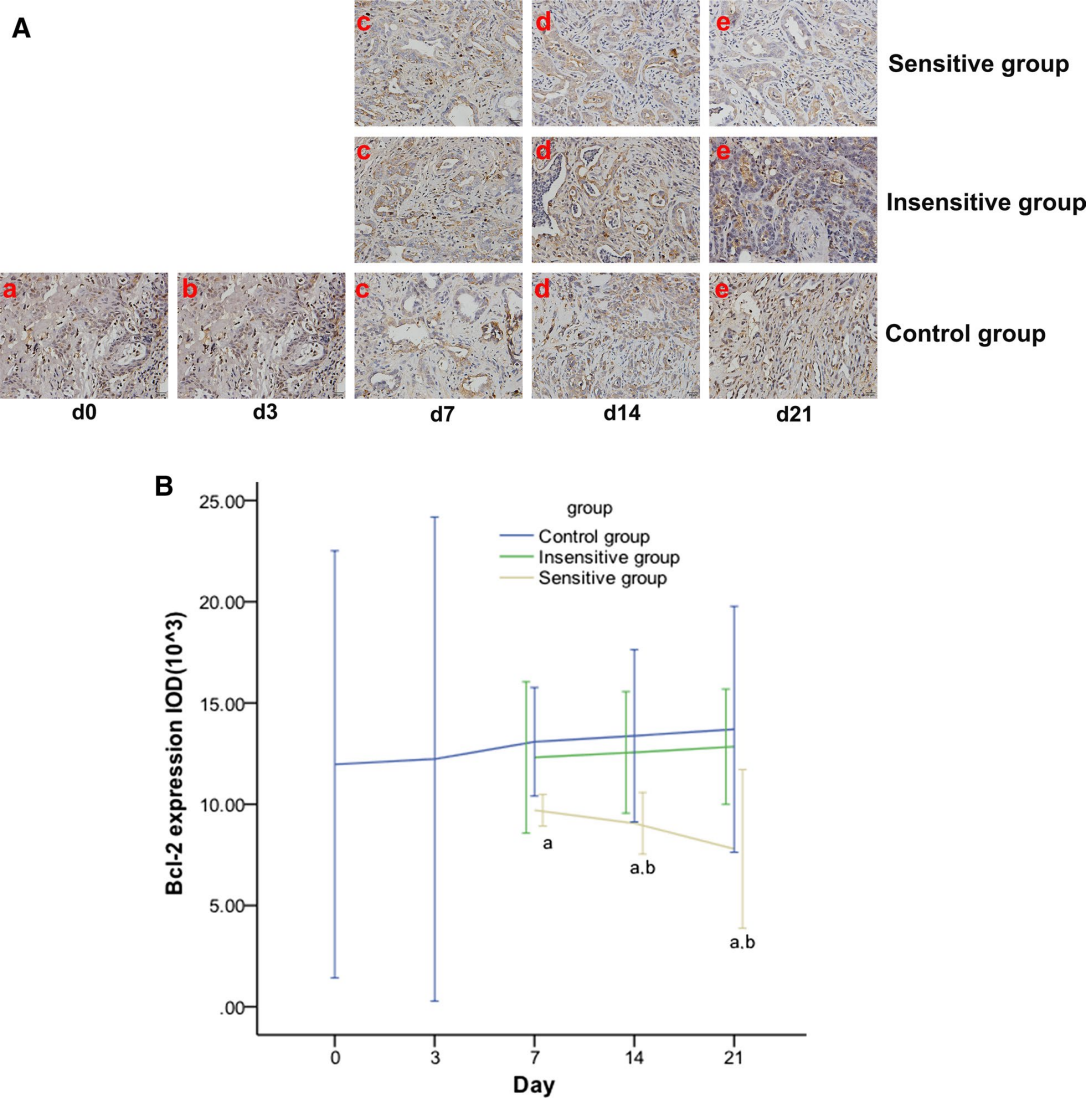
Effect of DTX on Bcl-2 expression in rat EOCs. IHC staining (HRF × 200) on day 0 (a), day 3 (b), day 7 (c), day 14 (d), and day 21 (e) of Bcl-2 expression (**A**), which display as brownish yellow granules in the cytoplasm and intercellular spaces, were analyzed. The expression of Bcl-2 (**B**) was decreased in the sensitive group but increased in the insensitive or control group. From day 7, significant differences were found in Bcl-2 in the sensitive vs. control group and in the sensitive vs. insensitive group from day 14. *p *< 0.05, ^a^: sensitive vs. control; ^b^: sensitive vs. insensitive

Bcl-2 expression and Δ% of Bcl-2 expression were significantly different between the sensitive and control groups on day 7, and between the sensitive and control or insensitive groups on days 14 and 21 (Fig. 7 and Table 5).

**Table 5:** Change rates of Bcl-2 expression at different time points (%)

Group	Day 7	Day 14	Day 21
Sensitive group	− 18.94 ± 2.53^a^	− 24.34 ± 4.95^a,b^	− 34.88 ± 12.73^a,b^
Insensitive group	2.85 ± 9.81	4.93 ± 7.88	7.30 ± 7.47
Control group	9.31 ± 8.06	11.76 ± 12.80	14.45 ± 18.27

Δ%: change rate

*p *< 0.05

^a^Sensitive vs. control

^b^Sensitive vs. insensitive

### Effective DTX therapy increases tumor apoptosis and necrosis in rat EOCs

**Fig. 8:**
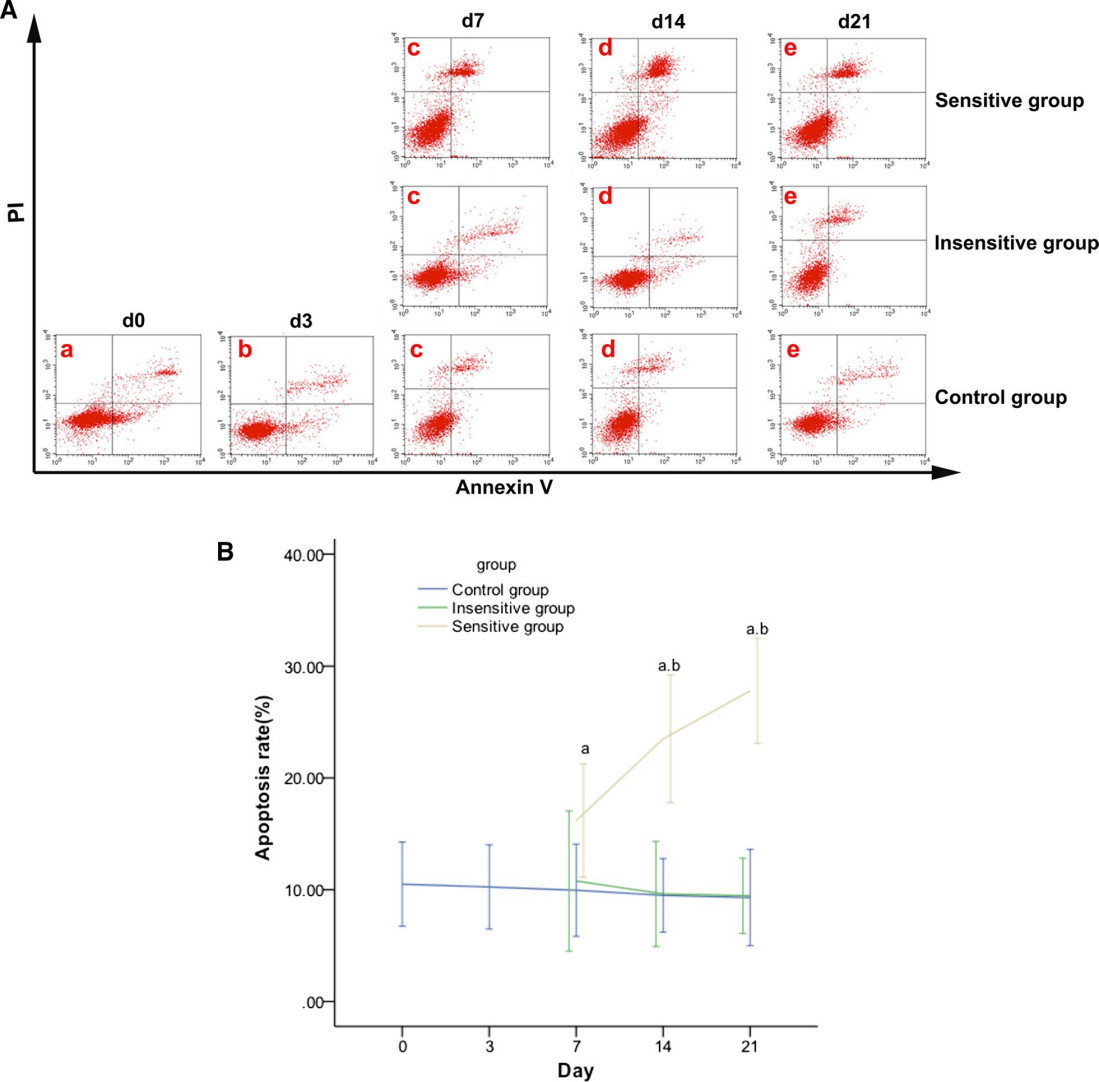
Effect of DTX on tumor apoptosis in rat EOC. On day 0 (a), day 3 (b), day 7 (c), day 14 (d), day 21 (e), apoptosis (**A**) was evaluated using Annexin V–fluorescein isothiocyanate/PI staining and flow cytometric analysis. Representative flow cytometry results: bottom right quadrant, cells stained primarily by Annexin V (early apoptotic cells); top right quadrant, cells stained by PI and Annexin V (late apoptotic cells); top left quadrant, cells stained primarily by PI (necrotic cells); bottom left quadrant, cells negative for Annexin V and PI. Apoptosis (**B**) was increased in the sensitive group but showed no remarkable change in the insensitive or control group. From day 7, significant differences were noted in apoptosis in the sensitive vs. control group and in the sensitive vs. insensitive group from day 14. *p *< 0.05, ^a^: sensitive vs. control; ^b^: sensitive vs. insensitive

**Fig. 9:**
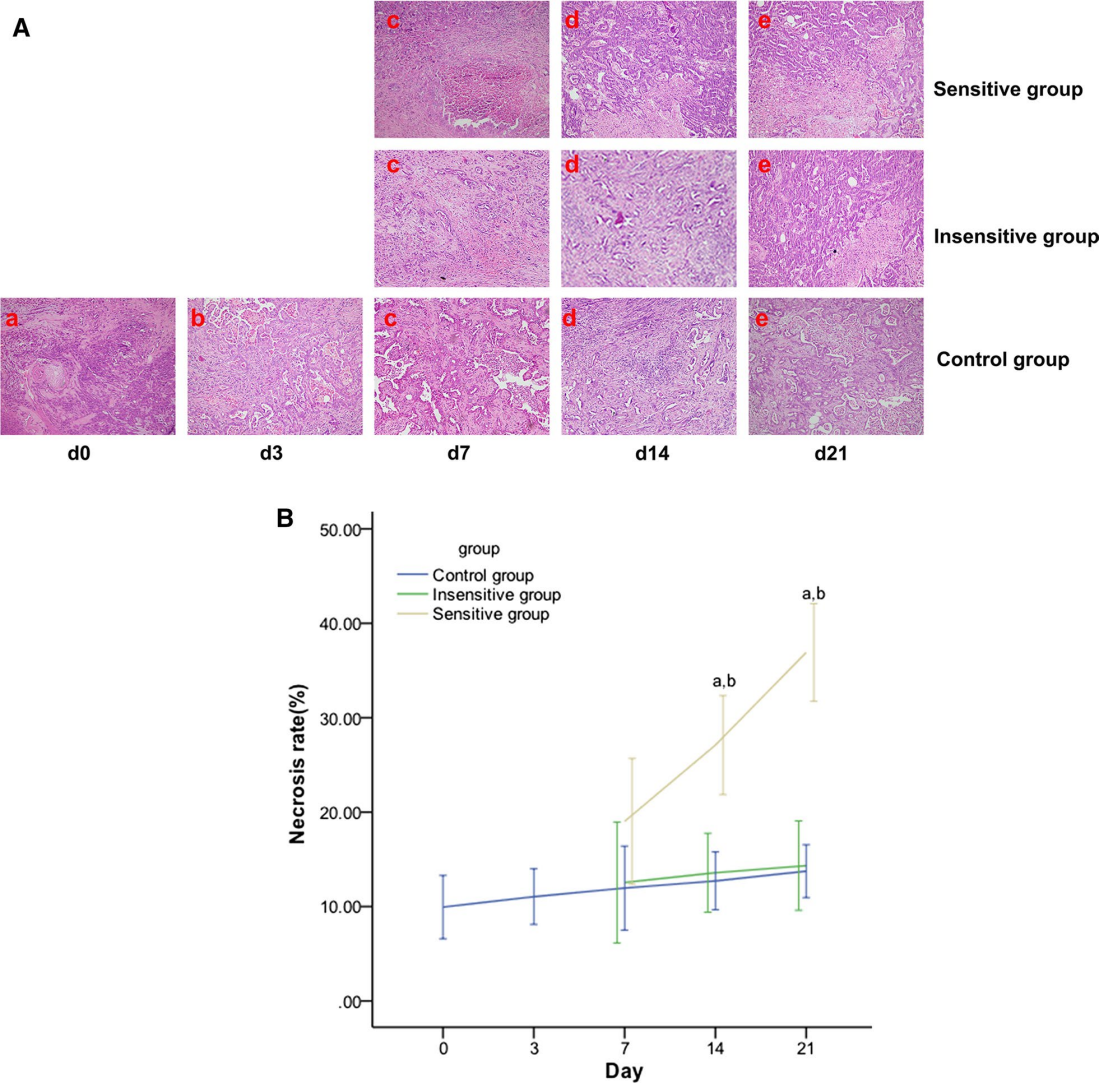
Effect of DTX on tumor necrosis in rat EOCs. Microscopic pictures (HRF × 200) on day 0 (a), day 3 (b), day 7 (c), day 14 (d), and day 21 (e) of the histopathology of EOC specimens (**A**), which are demonstrated as spotty or patchy necrosis scattering in the tumor, were analyzed. Tumor necrosis was increased in the sensitive group but showed no remarkable change in the insensitive or control group. On days 14 and 21, tumor necrosis (**B**) was significantly different among the three groups, except for that in the insensitive vs. control group. *p *< 0.05, ^a^: sensitive vs. control; ^b^: sensitive vs. insensitive

The apoptosis rate and Δ% of the apoptosis rate were significantly different between the sensitive and control groups on day 7, and between the sensitive and control or insensitive groups on days 14 and 21 (Fig. 8 and Table 6). The tumor necrosis and Δ% of necrosis lacked significant differences among the three groups on day 7 and showed significant differences between the sensitive and insensitive or control groups on days 14 and 21 (Fig. 9 and Table 6).

**Table 6:** Change rates of the apoptosis rate and tumor necrosis at different time points (%)

Time	Group	Δ% apoptosis rate	Δ% necrosis rate
Day 7	Sensitive group	54.12 ± 18.76^a^	91.36 ± 26.09
	Insensitive group	2.70 ± 18.76	26.22 ± 20.22
	Control group	− 5.17 ± 14.16	20.27 ± 16.10
Day 14	Sensitive group	123.91 ± 21.20^a,b^	172.62 ± 20.53^a,b^
	Insensitive group	− 8.28 ± 14.07	36.64 ± 13.21
	Control group	− 9.53 ± 16.18	27.88 ± 11.09
Day 21	Sensitive group	164.80 ± 17.52^a,b^	271.25 ± 20.21^a,b^
	Insensitive group	9.92 ± 10.08	44.14 ± 10.16
	Control group	− 11.43 ± 19.99	38.26 ± 10.16

Δ%: change rate

*p *< 0.05

^a^Sensitive vs. control

^b^Sensitive vs. insensitive

### Correlations between the change rates of DWI and DKI parameters and change rates of the tumor size, Ki-67, Bcl-2, apoptosis and tumor necrosis

The Δ% of K showed a highly positive correlation with the Δ% of Ki-67 and CA125, moderately positive correlation with the Δ% of tumor size and Bcl-2, and moderately negative correlation with the Δ% of apoptosis and tumor necrosis. The Δ% values of ADC and D were highly positively correlated with the Δ% values of Ki-67 and CA125, moderately positively correlated with the Δ% values of tumor size and Bcl-2, and moderately negatively correlated with the Δ% values of apoptosis and tumor necrosis (Table 7).

**Table 7:** Correlation between the Δ% of the DWI and DKI parameters and Δ% of the tumor size, Ki-67, CA125, Bcl-2, apoptosis and necrosis

	Δ% ADC		Δ% D		Δ% K	
	r	*p*	r	*p*	r	*p*
Δ% tumor size	− 0.546	< 0.001	− 0.558	< 0.001	0.594	< 0.001
Δ% Ki-67	− 0.788	< 0.001	− 0.810	< 0.001	0.812	< 0.001
Δ% CA125	− 0.697	< 0.001	− 0.672	< 0.001	0.751	< 0.001
Δ% Bcl-2	− 0.482	< 0.001	− 0.435	0.001	0.531	< 0.001
Δ% apoptosis	0.710	< 0.001	0.728	< 0.001	− 0.705	< 0.001
Δ% tumor necrosis	0.718	< 0.001	0.745	< 0.001	− 0.749	< 0.001

Tumor size: longest diameter; Δ%: change rate

## Discussion

Presently, DWI is the most widely used functional magnetic resonance imaging (fMRI) technique clinically. DKI is the development of DWI with high b values. Although rarely studied in animal models and clinical trials, DKI has shown its superiority in evaluating the efficacy of treatment for malignant tumors compared with DWI [22]. DWI and DKI can detect lesions by probing the random motion of water molecules in tissues and reflect the tumor cell density, cell membrane integrity and heterogeneity. Therefore, DWI and DKI can be applied theoretically to monitor the response to chemotherapy by quantifying the change in tumor microstructures.

Although RECIST is most commonly used to determine the curative effect of the tumor [23], morphology changes in the tumor are often not obvious and occur late. Our results demonstrated that neither the tumor size nor its change rate achieved a perfect result of an AUC of 1, a sensitivity of 100%, and a specificity of 100% to monitor the response of EOCs to DTX until day 21.

Wu et al. found a significantly increased ADC and D, and a decreased K in patients with cervical non-Hodgkin’s lymphoma who were sensitive to treatment at 7 days after chemotherapy [24]. Yu J et al. found that increased ADC and D were presented in patients with locally advanced rectal cancer who were sensitive to treatment after neo-adjuvant chemotherapy, and D was superior to ADC in evaluating treatment sensitivity [25]. However, DKI has not been used yet to evaluate the early response to radiation and chemotherapy. Our results demonstrated a significantly higher change rate of K, and lower change rates of D and ADC in the DTX-sensitive group than those in the insensitive and control groups from day 3 to day 21. However, differences in these parameters could not be found until day 7, 14 or 21. Early on day 7, the change rate of K had an AUC of 1, and sensitivity and specificity values of 100% and 100%, respectively, to detect the response to DTX using a cutoff value of 19.03% reduction in K. Compared with DWI and DKI parameters (K, D and ADC), the change rates of these parameters could reflect the changes in the tumor microstructures and function earlier, and more sensitively detect the response to chemotherapeutic agents in rat EOC. Additionally, the change rates of K, D and ADC could more effectively and earlier reflect the effective DTX treatment than the change rate of size and would be more reliable biomarkers to monitor the tumor response to chemotherapy and help make individualized therapy decisions.

ADC is closely correlated with tissue edema, necrosis, apoptosis and fibrosis, which reflect the internal structure and microenvironment of the tumor [26, 27]. Effective treatment can increase the ADC value of the tumor, and the increased ADC is correlated with tumor necrosis and apoptosis [28–30]. Based on the non-Gaussian distribution of water molecules, D is the corrected ADC and K is the mean kurtosis that reflects the deviation of diffusion from the Gaussian distribution. K increases with the complexity of the internal composition of the diseased tissue. A higher K indicates more complicated tumor microstructures. Our study indicated that effective DTX therapy could increase ADC and D, and decrease K. The explanation could be due to DTX inhibiting cell proliferation, inducing tumor necrosis and apoptosis [31–33], and consequently, increasing the extracellular space, movement of water molecules, ADC and D, and decreasing the complexity of the internal composition of the tumor and K [34].

Ki-67 is one of the most widely used markers for cell proliferation and malignancy [35]. CA125 is the clinically used biomarker for ovarian cancer and can reflect cell differentiation and progression of ovarian carcinoma, and can reflect the response to chemotherapy [36, 37]. Studies have shown that Ki-67-positive expression and the CA125 level play a significant role in the development, treatment and overall prognosis of EOC [38–40].

Previous studies have suggested that paclitaxel could reduce the Ki-67-positive expression and CA125 level of human ovarian carcinoma xenografts in nude mice [41, 42]. Our study in induced EOCs showed that K could effectively divide the DTX treatment group into the sensitive and insensitive groups on day 7. Ki-67-positive expression and the CA125 level were markedly decreased in the sensitive group compared with those in the insensitive group on day 7 and the control group on day 14. Furthermore, the change rates of Ki-67 and CA125 were negatively correlated with those of ADC and D and were positively correlated with the change rates of K. Our study suggested that ADC, D and K could noninvasively reflect the expression of tumor biomarkers in vivo and treatment-induced changes in the proliferative activity prior to changes in tumor morphology.

Bcl-2 contributes to stimulating cell proliferation and inhibiting apoptosis [43, 44]. A study by Wu H et al. showed that ADC was negatively correlated with Bcl-2 in rabbit soft-tissue VX2 carcinoma 5, 10, and 15 days after receiving radiotherapy [45]. Our study indicated that effective DTX could decrease Bcl-2 expression and induce the apoptosis of EOCs. Bcl-2 expression changes were negatively correlated with the changes in ADC and D and were positively correlated with the change of K. Apoptosis changes were positively correlated with the changes in ADC and D and were negatively correlated with the change in K. Additionally, our study indicated that treatment-induced apoptosis occurred prior to changes in the tumor size. We conjectured that the bursting of cell membranes and apoptosis relieved the movement limitation of water molecules, thus increasing ADC and D and decreasing K.

We also observed that, 14 days after DTX therapy, tumor necrosis was marked and occurred after changes in the DWI and DKI parameters (D, ADC and K). The change in necrosis was positively correlated with the changes in ADC and D and was negatively correlated with the change in K. Thus, we believe that tumor necrosis increases the extracellular space and movement of water molecules.

Our study had some limitations. First, we used a 3.0 T clinical MR scanner. A 7.0 T or higher field strength MR scanner will improve the image resolution. Second, respiratory gating should be used to improve the accuracy of DKI and DWI parameters.

## Conclusions

In conclusion, quantitative parameters of DWI and DKI, especially K, were superior to pathological and molecular biomarkers and imaging tumor size for the early detection and prediction of a response to DTX chemotherapy in EOC. Quantitative parameters of DWI and DKI could contribute to adjusting the treatment regimen for non-responders as soon as possible and improving the prognosis. However, it will take some time to apply the thought into large, randomized clinical trials or clinical application.

## Abbreviations

DWI: diffusion-weighted imaging
DKI: diffusion kurtosis imaging
DTX: docetaxel
EOC: epithelial ovarian carcinoma
DMBA: 7,12-dimethylbenz[a]anthracene
RECIST: response evaluation criteria in solid tumors
ADC: apparent diffusion coefficient
H&E staining: hematoxylin-eosin staining
IHC: immunohistochemical
CA125: cancer antigen 125
